# Immunological Perspective: *Helicobacter pylori* Infection and Gastritis

**DOI:** 10.1155/2022/2944156

**Published:** 2022-03-08

**Authors:** Hang Yang, Bing Hu

**Affiliations:** Department of Gastroenterology, West China Hospital, Sichuan University, Chengdu, Sichuan, China

## Abstract

*Helicobacter pylori* is a spiral-shaped gram-negative bacterium. Its infection is mainly transmitted via oral-oral and fecal-oral routes usually during early childhood. It can achieve persistent colonization by manipulating the host immune responses, which also causes mucosal damage and inflammation. *H. pylori* gastritis is an infectious disease and results in chronic gastritis of different severity in near all patients with infection. It may develop from acute/chronic inflammation, chronic atrophic gastritis, intestinal metaplasia, dysplasia, and intraepithelial neoplasia, eventually to gastric cancer. This review attempts to cover recent studies which provide important insights into how *H. pylori* causes chronic inflammation and what the characteristic is, which will immunologically explain *H. pylori* gastritis.

## 1. Introduction

*Helicobacter pylori* (*H. pylori*) has coevolved with its human host for no less than 30,000 years. It can safely colonize around the epithelium of gastric gland via their specific microstructure and self-synthesizing proteins such as chemoreceptors [1], flagella [2], and urease [3]. Chemotaxis assists *H. pylori* to find nutrients (urea and arginine) and avoid toxic substances like reactive oxygen species (ROS). *H. pylori* obtains nutrients from blood [4] and even extract nutrients from the host cells such as lipid, cobalt, iron, and nickel [5, 6]. Arginine is required for *H. pylori* growth and is sensed by TlpA [7]. Urea and gastric mucus pH gradient are sensed by TlpB for self-protection and chemotactic orientation [8, 9]. Additionally, *H. pylori* uses TlpB to sense injured sites and preferentially colonizes injured sites in the mouse stomach, independent on urea [10], which may indicate the inflammatory condition benefits the growth of *H. pylori*. Spiral shape is necessary to fast move and penetrate in the mucus layer via corkscrew-like movement [11]. Two flagellins (FlaA and FlaB) are indispensable for bacteria motility [12, 13]. FlaA mutants show a greater decline of motility than that of FlaB mutants [14]. *H. pylori* lacking MotB is nonmotile and retains only a nonfunctioning flagellar structure [13]. Urease is a nickel-dependent metalloenzyme [15]. Both insufficient cytoplasmic nickel availability or excessive nickel entry impair the activation of urease and the survival of *H. pylori* [16, 17]. Urea hydrolyzed by urease finally produces ammonia and CO_2_ to participate in lowering pH and the regulation of mucus viscoelasticity [18]. *H. pylori* enters the mucus layer after a short duration within the lumen and persistently distributes within approximately 25-30 *μ*m of mucosal epithelial cells where the pH ranges from 4.5 to 6.5, or directly on the epithelium or deep inside the glands [3, 19, 20]. After BabA/SabA-mediated adhesion [21], *H. pylori*, gastric epithelial cells, and leukocytes interact with each other to achieve the balance. During *H. pylori* infection, immune cells infiltrate to the lamina propria and submucosa, aiming to clear *H. pylori* [22]. *H. pylori* skews host immune response to avoid clearance and achieve persistence, such as cholesterol glycosylation, escaping the Toll-like receptor (TLR) recognition, tolerating dendritic cells (DCs), blocking T cell proliferation, inducing Treg skewing, and upregulating PD-L1 [23–26].

Inflammation is triggered when innate immune cells detect infection or tissue injury [27]. Although *H. pylori* manipulates the host immune system, inadequate immune response and inflammation are still initiated, leading to chronic active gastritis. Inflammation is an essential and a complex biological process that protects the body from potential harm caused by infection or injury [28] and develops in response to pathogen-associated molecular patterns (PAMPs) from *H. pylori* and damage-associated molecular patterns (DAMPs) from damaged epithelial cells [29]. Pattern recognition receptors (PRRs) are membrane associated or soluble, which are owned by both immune and nonimmune cells, and respond to PAMPs and DAMPs, initiating downstream signaling cascades, including the production and secretion of pro- and anti-inflammatory cytokines to further modulate immune response [30].

## 2. PAMPs Derived from *H. pylori* Inducing Inflammation: NF-*κ*B and Type I IFNs

### 2.1. Antigen-Presenting Cells (APCs)

PRRs expressed on APCs (macrophages, DCs, and B cells) including TLRs, nucleotide-binding oligomerization- (NOD-) like receptors (NLRs), and C-type lectin receptors (CLRs) can detect PAMPs derived from *H. pylori* [31] such as lipopolysaccharide (LPS), lipoproteins and peptidoglycan (TLR2), dsRNA and polycytidylic acid (TLR3), LPS and heat shock proteins (TLR4), flagellin (TLR5 and 11), and unmethylated CpG containing ssDNA (TLR9) [32–34]. TLR activation increases the activity of NF-*κ*B and transcription of type I IFNs [35]. Both TLR2 and TLR4 on DCs and macrophages can recognize LPS and shape the *H. pylori*-induced pro- and anti-inflammatory cytokines and chemokine milieu [36, 37]. Receptor complex combining TLR2 with TLR1, 6 or 10, not TLR4 is also reported [38–40], which further indicates both pro- and anti-inflammation roles of TLR2. TLR9 is expressed exclusively in intracellular vesicles and recognizes unmethylated CpG DNA motifs to induce the transcription of NF-*κ*B and IRF7 via MyD88 and subsequent increase of inflammatory cytokines and type I IFNs in APCs [41, 42]. It has also demonstrated that TLR9 is involved in both suppressing and promoting inflammation after recognizing *H. pylori* DNA [41]. For example, increasing Th1 cells (IFN*γ*) and Th17 cells (Th17) is found in *Tlr9−/−* mice [43, 44], and the roles of activating NF-*κ*B, upregulating the expression of COX-2/prostaglandin E2, and activating neutrophils are also found after activating TLR9 [45, 46]. CLRs expressed by DCs are pivotal for both antigen presentation and Th cell differentiation [47]. CLRs on DCs and macrophages can be activated by *H. pylori* metabolites modified from host cholesterol (cholesteryl acyl *α*-glucoside and cholesteryl phosphatidyl *α*-glucoside), exacerbating gastritis [48, 49]. C-type lectin DC-specific intercellular adhesion molecule-grabbing nonintegrin (DC-SIGN) can recognize LPS Le antigens [50], which can be misused by distinct mechanisms that either circumvent antigen processing or alter TLR-mediated signaling to decrease Th1 cells and increase Treg cells [51, 52]. For example, *H. pylori* modulates the Th1/Th2 balance through the phase-variable interaction between LPS and DC-SIGN and the variation of O-antigen decorated by fucose residues binding to DC-SIGN to block Th1 development [53, 54]. B cells also express TLR and MyD88. CpG (TLR9), LPS (TLR4), and peptidoglycan (TLR2) are found to induce B cell-derived IL-6, IL-12, and IL-10 [55, 56]. TLR signaling in B cells inhibits inflammatory T cell response (both Th1 and Th17 cells), which can be controlled by TLR agonists [57]. Further B cells produce IL-10 after being activated by *H. pylori* and suppress the differentiation of DCs activated by *H. pylori* [58]. And activated TLR2 by *H. pylori* on B cells also induces Treg (IL-10) [59].

### 2.2. Gastric Epithelial Cells

TLR2, TLR4, TLR5, TLR9, and NOD1 are expressed by epithelial cells and induce NF-*κ*B activation and IFN production in these cells to defense against *H. pylori*, as a central principle of mucosal immunity [60–63]. Increasing expression of TLR2, TLR4, TLR5, and TLR9 in gastric epithelia of children' gastritis [64]; TLR4, TLR5, and TLR9 in adults' gastritis [60]; TLR2, TLR4, and TLR5 in gastric dysplasia [65]; and TLR4, TLR5, and TLR9 in gastric cancer (GC) [61] is found. Expression of TLR2 and TLR4 in chronic gastritis caused by *H. pylori* remains increased after eradication therapy in 3 months [66]. TLR2 is the most extensively expressed receptor among all the TLRs in gastric mucosa infected by *H. pylori* [67, 68]. In gastric epithelial cells, TLR2 cooperates with TLR4 to strengthen the innate immune response to LPS and activate NF-*κ*B and inducible nitric oxide synthase (iNOS) [68]. Significantly increasing TLR9 in gastric epithelial cells are demonstrated in patients residing in the region with a high GC risk, and *H. pylori* isolated from them can cause increasing activation of TLR9 [69]. Regarding on CLRs, besides of the expression on the surface of DCs, DC-SIGN is overexpressed in gastric epithelial cells, when facing LPS stimulation [70, 71] and induces a Th1 dominating cytokine response [71]. Furthermore, DC-SIGN stimulated by LPS interacts with TLR4, promotes NLRP3, and regulates the production of IL-1*β* and IL-18 in gastric epithelial cells [70]. *H. pylori* peptidoglycan delivered into host cells by the T4SS is recognized by epithelial cells via Nod1 [72], leading to NF-*κ*B activation and the production of *β*-defensin and type I IFNs from Nod1-activated gastric epithelial cells [73–75]. *H. pylori* can secrete outer membrane vesicles (OMVs) separated from the bacterial outer membrane [76]. OMVs containing peptidoglycan enter epithelial cells at cholesterol-rich lipid rafts and induce NOD1-dependent response [77]. Apart from PPRs, D-glycero-*β*-D-manno-heptose 1,7-bisphosphate (*β*-HBP) is a T4SS-dependent effector of NF-*κ*B activation via alpha-protein kinase 1- (ALPK1-) TRAF-interacting protein with forkhead-associated domain (TIFA) in gastric epithelial cells [78]. ADP-glycero-*β*-D-manno-heptose (*β*-ADP heptose), a derivative of *β*-HBP, is more active than *β*-HBP [79]. *β*-ADP heptose mediates NF-*κ*B activation and cytokine expression after directly binding the N-terminal domain of ALPK1 [80]. Additionally, CagA also contributes to the NF-*κ*B signaling after TIFA and NOD1 activation [81].

## 3. DAMPs from Gastric Epithelium Inducing Inflammation

Acute and chronic inflammation caused by *H. pylori* can damage epithelial cells and induce inflammatory edema, atrophy, and necrosis/apoptosis. DAMPs are released during tissue damage and typically derived from intracellular and extracellular continents, which are recognized by PRRs (TLRs and NLRs) and by non-PRRs (RAGE, CD44, integrins, and CD91) to recruit neutrophils and monocytes and activate inflammation and tissue repair [82]. Interleukin-1 (IL-1) and tumor necrosis factor (TNF) are the notable proinflammatory cytokines in this process.

IL-1 (IL-1*α* and IL-1*β*) is the proinflammatory cytokine mainly produced by macrophages and acts through IL-1 receptor (IL-1R), which is important to recruit neutrophils and monocytes and to induce additional proinflammatory cytokines [83]. Caspase-1 is responsible to cleave IL-1*β* into the biologically active form [84]. Caspase-1 is activated by inflammasomes composed of a PRR of the NLR family such as NLRP3, NLRC4, and AIM2 inflammasome [85]. Research has identified that *H. pylori* can activate inflammasomes. For example, caspase-1 activation and production of IL-1*β* and IL-18 in mice are the consequence of *H. pylori* infection, in which IL-1*β* is produced for clearance and IL-18 is for persistence [86]. AIM2 inflammasome is demonstrated to recognize cytoplasmic DNA [87], which is also induced by the OMVs of gram-negative pathogens [88]. NLRC4 expression is upregulated by proinflammatory stimuli like TNF*α* [89]. *H. pylori* exploits the NLRC4 inflammasome to enhance neutrophil infiltration and induce IL-18 production in gastric epithelial cells to block *β*-defensin expression via NF-*κ*B activation [90]. NLRP3 can recognize nucleic acids, bacterial proteins, and metabolites [91]. The proinflammatory cytokines released upon NLRP3 activation are IL-1*β*, IL-18, HMGB1, leukotrienes, and prostaglandins [92]. *H. pylori* infection activates the NLRP3 inflammasome and IL-1*β* production in neutrophils [93, 94], differentiated macrophages [95], and DCs [96]. Furthermore, the activation of NLRP3 inflammasome by *H. pylori* via ROS signaling pathway also leads to the production of IL-1*β* and IL-18 in human monocytes [97]. These results suggest a dual role of the inflammasome in *H. pylori* infection. Additionally, Treg cells can be activated by the activation of axis of urease enzyme of *H. pylori*/TLR2/NLRP3/caspase-1/IL-18 [98]. Furthermore, the application of exogenous activators induces NLRP3 inflammasome formation, and the secretion of high amounts of IL-1*β* in infected cells, which indicates cellular injury regardless of causes (e.g., bile, smoking, alcohol, drugs, and other gastric microbiota), may have synergistic effect with *H. pylori* infection to exacerbate damage to gastric epithelium [99].

TNF has been participated in regulating immunity and inflammation. It is widely and constitutively expressed by activated immune cells, as well as by fibroblasts and endothelial and epithelial cells responding to proinflammatory cytokines including TNF itself [100, 101]. It is cleaved by TNF*α*-converting enzyme to release soluble TNF*α* [102]. TNF mediates inflammatory pathology through binding to TNFR1 and TNFR2 [103]. TNFR1 interacts strongly with both membrane and soluble TNF*α*, whereas TNFR2 binds to membrane TNF*α* with much higher affinity. TNFR1 is expressed by near all cells. TNFR2 expression is limited to cells of immune and endothelial origin [101]. It means *H. pylori* infection can induce TNF production and activate its signal pathways in many aspects. TNF downstream pathways mainly involve NF-*κ*B, MAPKs, caspases, and ROS/RNS. TNFR1 and 2 induce the activation of MAPK and NF-*κ*B. TNFR1 can also stimulate apoptosis and necroptosis, as it harbors a death domain in the cytoplasmic part [101, 104]. As a proinflammatory cytokine, TNF*α* causes vasodilatation and edema, leukocyte adhesion to epithelium, and oxidative stress in inflammatory sites, mediated by the induction of NO, prostanoids, and ROS [105, 106]. TNF*α* may also regulating the production of ROS and RNS [107]. Macrophages, DCs, and gastric epithelial cells produce TNF and IL-1*β* in a dose- and time-dependent manner after exposure to *H. pylori* [108–110]. TNF*α* induces the apoptosis of parietal cells in *H. pylori* infected rat [111]. Increasing TNF*α* is found in *H. pylori* infected chronic atrophic gastritis (CAG) and associated with chronic inflammation degree [112]. Soluble TNF receptors (sTNFRs) are shown actively produced in *H. pylori* infected gastric mucosa, and anti-sTNFR monoclonal antibodies increase TNF-induced gastric epithelial cell apoptosis, which suggests that sTNFR has a protective effect [113]. In addition, TNFR1 increase is seemly related to the aggressiveness of gastric lesions [114]. These may indicate severe infection cause increasing TNF*α* companied with increasing sTNFRs to avoid more loss of gastric epithelial cells.

NF-*κ*B activation may prevent cell death because it controls the transcription of a number of genes involved in cell survival, proliferation, and inflammation [107]. Oxidative stress has a crosstalk with both TNF [107] and NF-*κ*B [115] via bidirectional effects, as they have complex interaction with each other during production process. Briefly, it means oxidative stress can both activate and inhibit NF-*κ*B pathway, and NF-*κ*B pathway has anti- and prooxidant role in oxidative stress [116]. TNF promotes the production of ROS and NF-*κ*B. TNF-induced ROS inhibits NF-*κ*B activation, reduces NF-*κ*B-mediated survival signaling, and explains the cell death associated with high ROS levels [116]. Mitochondrial ROS can facilitate TNF-mediated NF-*κ*B activation [115]. Furthermore, these effects are further complicated by TNF-induced NO production. TNF-induced NF-*κ*B promotes the transcription of the gene that encodes iNOS for producing NO with anti- and prooxidant roles [117]. There may be also a subtle NO/ROS/RNS balance in TNF signaling [118]. *H. pylori* increases ROS and RNS mainly from immune cells such as neutrophils and gastric epithelial cells, correlating with the severity of mucosal inflammatory damage and genetic instability [119, 120]. In addition, iNOS expression is highly induced in the epithelium of atrophic gastritis, as well as metaplasia and dysplasia [121]. NF-*κ*B can stimulate iNOS in gastric epithelial cells maybe further to prevent cell death [68], which depends on DNA damage caused by ROS/RNS in gastric epithelial cells [122, 123]. Furthermore, the long-lasting state with inflammation, oxidative stress, and DNA damage may also lead in GC [124].

## 4. Neutrophil Recruitment and Inflammation

Neutrophils are the first leukocytes recruited to the inflammatory site during acute inflammation, aiming at eliminating pathogens by phagocytosis, deregulation, and neutrophil extracellular traps. Neutrophils and macrophages produce ROS, proteases, and growth factors, leading to tissue destruction, fibroblast proliferation, abnormal accumulation of collagen, and fibrosis [125]. For example, ROS produced by neutrophils and NO produced by macrophages fail killing *H. pylori*, but do damage on gastric epithelial cells (e.g., nuclear DNA and mitochondria, even cell death or gastric carcinogenesis) [119, 124, 126]. Neutrophil recruitment is started by changes on endothelial surface induced by inflammatory cytokines released from tissue-resident sentinel leukocytes exposed to pathogens [127]. Macrophages and mast cells reside in tissues are sentinel cells to initiate neutrophil recruitment via increasing the permeability of local blood vessels and chemokine secretion after the activation of PRRs such as TLRs, NLRs, and CLRs [128, 129]. For instance, TNF*α* produced from activated macrophages and mast cells acts as a crucial role to recruit neutrophils [130]. *H. pylori* infection is characterized by rapid and continuous recruitment of neutrophils followed by T and B cells, plasma cells, and macrophages [131]. Apart from the activation of PPRs causing neutrophil recruitment (e.g., both TNF*α* and IL-1*β* can induce neutrophil recruitment), *H. pylori* neutrophil-activating protein (HP-NAP) can induce trans-endothelial migration of neutrophils and activate neutrophils such as the release of myeloperoxidase and production of ROS/RNS, IL-8, and CCL4 [132–135], which is attenuated when lacking SabA [136] or ablating hepatoma-derived growth factor [137]. HP-NAP is shown to determine the host risk of dyspepsia by ROS exposure and chronic inflammation [138]. Additionally, HP-NAP has immune modulating roles and induces cytokines from other immune cells [139]. After entering into the inflammatory tissue site, neutrophils express many cell surface receptors [140] and recognize PAMPs and DAMPs by neutrophil PRRs [141], opsonins by opsonic receptors and bacterial products [142], and endogenous molecules released during inflammation by G protein coupled receptors [143]. Neutrophils are involved in the complex bilateral interactions with aforementioned immune cells [144, 145]. HP-NAP can regulate immunity, which may be attributed to the regulation from neutrophil itself, which means immunity is actually regulated by neutrophils, and HP-NAP is just responsible to recruit neutrophils.

## 5. Adaptive Immune and Inflammation

Adaptive immunity is responsible for the production of antibodies and the activation of cytotoxic lymphocytes after recognizing antigen peptides presented by APCs [146]. Naïve CD4+ T cells are induced to differentiate towards Th1 (IL-2 and IFN*γ*), Th2 (IL-4, IL-5, IL-3, and IL-13), Th17 (IL-17 and IL-22), and Treg (IL-10 and TGF-*β*) phenotypes according to the local cytokine milieu [147, 148]. *H. pylori* infection can induce these cells differentiation with different proportions and a balanced cytokine network. As a pathogen, innate and subsequent adaptive immune responses are evoked to eliminate *H. pylori*. Th1 and Th17 and their corresponding cytokines are required for infection control [149, 150]. As a persistent colonizer coevolving with human, it can skew adaptive immune response. For example, both VacA and *γ*-glutamyltransferase (GGT) possess pro- and anti-inflammatory effects. They block the proliferation of T cells via blocking the cell cycle [151, 152] and induce cell death and proinflammatory cytokine production (TNF-*α* and IL-1*β* induced by VacA, and cyclooxygenase-2, prostaglandin E2, NF-*κ*B, and IL-8 induced by GGT) [153–157]. *H. pylori* can induce the Treg cell differentiation and increase IL-10 and TGF-*β*1 in infected patients, in particular in children [158–160]. Treg cell differentiation requires the direct interaction between naïve T cells and tolerogenic DCs exposed to *H. pylori* [161, 162]. GGT and VacA contribute to *H. pylori*'s tolerance-promoting effects on DCs [163]. The cytokine network is concluded in detail.

IL-12 cytokine family contains IL-12, IL-23, IL-27, and IL-35, which serve as a vital bridge between innate and adaptive response [164]. IL-12 expression regulates innate response and controls the differentiation of Th cell type. IL-12 is produced by DCs, monocytes/macrophages, and neutrophils and triggers the differentiation of Th1 cells (IL-2 and IFN*γ*) [165]. IL-12 can also activate NK cells, other T cells, and DCs/macrophages to produce IFN*γ* [166, 167]. IFN*γ* stimulates the bactericidal activity of phagocytic cells to boost innate immune response, which is modulated by IL-4, IL-10, and IL-18 [168]. IL-23 is produced by myeloid cells, especially DCs and monocyte/macrophage lineage cells [169]. IL-23 induces IL-1*β* and TNF*α* produced by myeloid cells and NK and T cells and drives IL-12 and IFN*γ* production [168]. IL-23 combined with TGF-*β* and IL-6 determines the differentiation of Th17, in which TGF-*β* plus IL-6 (mainly produced by monocytes/macrophages) act as the differentiation factor and IL-23 acts as the growth and stabilization factor [170]. Rapid IL-23/IL-17 immune response can promote chronic inflammation with cytokines such as IL-17, IL-6, IL-8, and TNF [171]. For example, activated sentinel DCs and macrophages produce IL-23 to trigger the release of IL-17 from tissue-resident T cells and NK cells. IL-17 increases the secretion of IL-1, IL-6, IL-8, CXC ligand 1, and TNF in stromal, epithelial, and endothelial cells and monocyte subpopulations and further recruits neutrophils [172]. IL-27 produced by the myeloid lineage (mainly monocytes and DCs) [173] limits the production of IL-2 and GM-CSF; reverses the IL-23 mediated lineage commitment of Th17 cells; induces IL-10 produced from Th1, Th2, Th17, and Treg cells; and promotes Treg cells specialized to limit Th1 cells [174]. Further, both IFN*γ* and IL-27 promote a population of Treg cells, which restrict Th1 cell-mediated pathology [175].

IL-35 is produced by Treg cells, CD8+ Treg cells, DCs, and B cells. It inhibits Th17 cell differentiation and promotes both Treg cell proliferation and corresponding functions [176, 177]. IL-10 is produced by APCs, mast cells, eosinophils, neutrophils, NK cells, and T cells [178]. IL-10 has profound anti-inflammatory functions. IL-10 inhibits the release of proinflammatory cytokines (e.g., TNF*α*, IL-1*β*, IL-6, IL-8, and GM-CSF) and chemokines (e.g., MCP1, IL-8, and IP-10) from DCs and monocytes/macrophages. IL-10 suppresses both IL-12 and IL-23 to limit CD4+ T cell differentiation and proliferation. IL-10 attenuates neutrophil recruitment by decreasing inflammatory cytokines [179]. TGF-*β*1 is widely expressed in leukocytes and stromal cells and responsible for wound healing, immune tolerance, and the modulation of cell growth and differentiation [180]. TGF-*β*1 combined with IL-2 is critical for the differentiation of Treg cells from naïve T cells [181]. TGF-*β*1 produced by Treg cells is required to inhibit Th1 cell differentiation and promote immune tolerance [182, 183].

Type I IFNs (IFN*α* and IFN*β*) and type II IFN (IFN*γ*) orchestrate innate and adaptive immunity via multiple mechanisms. Type I IFNs are produced by almost every cell type with DCs, macrophages, and epithelial cells mainly through TLR-dependent pathways [184, 185]. Type I IFNs activate NK cells, macrophages, and DCs to boost innate immunity [186] and enhance adaptive immunity through promoting the differentiation of T and B cells and their activation. Type I IFNs can also inhibit Th17 cells and induce IL-10 and PD-1 ligand expression by immune cells like DCs and macrophages in chronic infection [187, 188]. IFN*γ* is the sole type II IFN, exclusively produced by Th1 cells, CD8+ cytotoxic T lymphocytes (CTLs), NK cells, innate lymphoid cells (ILCs), and DCs [189]. An early production of IFN*γ* is from ILCs, and abundant and sustained IFN*γ* are produced by Th1 cells or CTLs after recognizing microbial peptides from APCs [190]. IFN*γ* is positively regulated by IL-12 and IL-18 and negatively regulated by IL-4, IL-10, TGF-*β*, and glucocorticoids [189]. The effects of IFN*γ* on adaptive immunity have been reviewed on facilitating Th1 cell differentiation, inhibiting Th2 and Th17 cells, activating Treg cells, and promoting B cell class switching [191]. IFN*γ* can also strongly promote innate immune. IFN*γ*-polarized macrophages are highly responsive to a variety of inflammatory stimuli such as TNF, type I IFNs, microbial products, and ligands for TLRs [192]. IFN*γ* orchestrates the differentiation of monocytes into DCs and macrophages, which is the primary sources of IL-12 at the infection site [193].

## 6. Conclusions

*H. pylori* gastritis is ultimately attributed to the activation of PRRs on APCs, gastric epithelial cells, and neutrophils. And complex multilateral crosstalk between gastric epithelium, innate immune response, and adaptive immune response permit *H. pylori* persistent colonization. From the aforementioned immune response and cytokines, there is a dynamic balance between inflammation and immunity, which processes to a newer and severer balance gradually, as the persistence of *H. pylori* provides sustained stimuli. The balance is achieved by pro- and anti-inflammatory cytokine network from these leukocytes. It further indicates that relieving or aggravating the inflammation by increasing/decreasing one specific cytokine to break this balance may facilitate the eradication of *H. pylori*, which also explains the reason why addition of vitamin C (an antioxidant and -inflammation role to break the balance) to *H. pylori* eradication treatment may increase the eradication rate. Further research is needed.
